# 

**Published:** 1910-12-24

**Authors:** 


### CONTENTS.

| | PAGE |
|---|---|
| **Humanitarianism** ... ... ... ... ... ... ... ... | 367 |
| **Colonial Drugs** ... ... ... ... ... ... ... ... | 368 |
| **Annotations—** | |
| The late Professor Mosso—Health Resorts and Gambling—The Legal Responsibility of Opticians ... ... ... ... ... | 369 |
| **By the Way** ... ... ... ... ... ... ... ... | 370 |
| **Special Articles—** | |
| The Practitioner: Reputed and Actual ... ... ... ... | 373 |
| A Medical Worthy of the Early Eighteenth Century ... ... | 374 |
| The Stethoscope ... ... ... ... ... ... ... ... | 375 |
| **Practical Notes on Diagnosis and Treatment** ... | 378 |
| **News and Events of the Week** ... ... ... ... | 379 |
| **Parliament and Publications** ... ... ... ... ... | 380 |
| **The Week in the Courts** ... ... ... ... ... | 380 |
| **Appointments and Resignations** ... ... ... ... | 380 |
| **Obituary** ... ... ... ... ... ... ... ... | 380 |
| **The Practitioner's Relaxations—** | |
| The Compilation of a Local History ... ... ... ... | 381 |
| Nature Notes—VIII. ... ... ... ... ... ... | 382 |
| **The Week's Work—** | |
| Mr. Silver's Bequests—Christmas and the Hospitals—An Out-Patient Department Inquiry—Anæsthetic Fatalities—The Open Ether Method—Incorporated Association of Hospital Officers—This Week's Drug Market ... ... ... ... ... | 383 |
| **Special Institutional Article—** | |
| The Newcastle Infirmary Resignations ... ... ... ... | 385 |
| **Gifts and Bequests** ... ... ... ... ... ... ... | 386 |
| **Jottings** ... ... ... ... ... ... ... ... ... | 386 |
| **The League of Mercy** ... ... ... ... ... ... ... | 387 |
| **Metropolitan Hospital Sunday Fund** ... ... ... ... | 391 |
| **Hospital Medical Schools and the King's Fund** ... | 394 |
| **Editor's Letter-Box—** | |
| Meals for Necessitous School Children during the Christmas Holidays ... ... ... ... ... ... ... ... | 394 |
| **The Hospital World** ... ... ... ... ... ... ... | 395 |
| **Institutional Notes and News** ... ... ... ... ... | 396 |

